# Targeting hormone-resistant breast cancer cells with docetaxel: a look inside the resistance

**DOI:** 10.20517/cdr.2022.96

**Published:** 2023-02-07

**Authors:** Alexander M. Scherbakov, Anna A. Basharina, Danila V. Sorokin, Ekaterina I. Mikhaevich, Iman E. Mizaeva, Alexandra L. Mikhaylova, Tatiana A. Bogush, Mikhail A. Krasil’nikov

**Affiliations:** ^1^Department of Experimental Tumor Biology, Blokhin N.N. National Medical Research Center of Oncology, Moscow 115522, Russian Federation.; ^2^Group of Molecular Tumor Markers, Blokhin N.N. National Medical Research Center of Oncology, Moscow 115522, Russian Federation.

**Keywords:** Cancer, docetaxel, 4-hydroxytamoxifen, class III β-tubulin, resistance, breast cancer, estrogen receptors alpha

## Abstract

**Aim:** The study aims to analyze the effect of long-term incubation of ERα-positive MCF7 breast cancer cells with 4-hydroxytamoxifen (HT) on their sensitivity to tubulin polymerization inhibitor docetaxel.

**Methods:** The analysis of cell viability was performed by the MTT method. The expression of signaling proteins was analyzed by immunoblotting and flow cytometry. ERα activity was evaluated by gene reporter assay. To establish hormone-resistant subline MCF7, breast cancer cells were treated with 4-hydroxytamoxifen for 12 months.

**Results:** The developed MCF7/HT subline has lost sensitivity to 4-hydroxytamoxifen, and the resistance index was 2. Increased Akt activity (2.2-fold) and decreased ERα expression (1.5-fold) were revealed in MCF7/HT cells. The activity of the estrogen receptor α was reduced (1.5-fold) in MCF7/HT. Evaluation of class III β-tubulin expression (TUBB3), a marker associated with metastasis, revealed the following trends: higher expression of TUBB3 was detected in triple-negative breast cancer MDA-MB-231 cells compared to hormone-responsive MCF7 cells (*P *< 0.05). The lowest expression of TUBB3 was found in hormone-resistant MCF7/HT cells (MCF7/HT < MCF7 < MDA-MB-231, approximately 1:2:4). High TUBB3 expression strongly correlated with docetaxel resistance: IC_50_ value of docetaxel for MDA-MB-231 cells was greater than that for MCF7 cells, whereas resistant MCF7/HT cells were the most sensitive to the drug. The accumulation of cleaved PARP (a 1.6-fold increase) and Bcl-2 downregulation (1.8-fold) were more pronounced in docetaxel-treated resistant cells (*P *< 0.05). The expression of cyclin D1 decreased (2.8-fold) only in resistant cells after 4 nM docetaxel treatment, while this marker was unchanged in parental MCF7 breast cancer cells.

**Conclusion:** Further development of taxane-based chemotherapy for hormone-resistant cancer looks highly promising, especially for cancers with low TUBB3 expression.

## INTRODUCTION

Breast cancer is considered a major cause of death from cancer in women around the world^[1]^. Breast cancer has several various molecular subtypes, and more than 65%-70% of breast tumours are hormone receptor-positive (ERα+)^[2]^. ERα-positive cancers are initially driven by hormone activation of estrogen receptor α, which in turn, induces pro-proliferative/pro-oncogenic cascades^[3]^. This fact explains the effectiveness of therapies that target the hormone molecular pathway in ERα-positive breast cancer^[4]^. The first antiestrogen for ERα-positive breast cancer treatment was tamoxifen, a selective estrogen receptor modulator, inhibiting the activity of the estrogen receptor alpha (ERα)^[5,6]^. The mechanism of its action in breast cells is the competition with endogenous estrogens for binding estrogen receptor α; thus, tamoxifen inhibits the estrogen-driven, pro-proliferative transcription program in breast cancer cells^[6,7]^ and also activates G-protein-coupled ER (GPER1)^[8]^. In some cases, long-term tamoxifen treatment leads to the development of resistance, cellular mechanisms of which are complex and not fully clear. They include regulation of ESR1 expression by epigenetic factors^[9]^, mutations of ESR1^[10]^, alternative splicing events^[11]^, alterations in the hormone-binding domain^[10]^, differential recruitment of coregulators^[12]^, factors of the tumor microenvironment^[13]^ and many others ^[14,15]^. One of the major mechanisms of the development of hormone resistance is dysregulation of the PI3K/AKT/mTOR pathway that cross-talks with estrogen-mediated signaling^[16]^. Inhibition of the PI3K/AKT/mTOR pathway results in reduced cell proliferation and survival, but this activates compensatory mechanisms that confer resistance to inhibitors. In several studies, it was shown that activation of the Akt pathway was associated with tamoxifen resistance in breast cancer cells, poor prognosis and decreased relapse-free survival, and increased incidence of relapse with distant metastases^[17-19]^.

βIII-tubulin is a well-known tubulin isotype. Monomers of α- and β-tubulin spontaneously assemble and polymerize to form the microtubules, cytoskeletal polymers involved in critical cellular processes such as mitosis, cell motility, and intracellular transport. Moreover, β-tubulins are GTPases as well and regulate the kinetics of microtubule assembly and disassembly^[20]^. βIII-tubulin is overexpressed in many tumours, including resistant tumours^[21,22]^, and is regulated by hormones^[23]^. Docetaxel, a taxane, is an antimicrotubule agent effective in the treatment of patients with breast cancer^[24]^. Researchers are conducting extensive investigation of this drug to improve treatment efficacy and delivery selectivity^[25-27]^. The study aims to analyze the effect of long-term incubation of ERα-positive breast cancer cells with 4-hydroxytamoxifen on their sensitivity to a tubulin polymerization inhibitor docetaxel.

## METHODS 

### Cell lines and compounds

The triple-negative MDA-MB-231 and hormone-dependent MCF7 breast cancer cell lines were purchased from ATCC collection. The cells were maintained in a standard DMEM medium (Gibco) supplemented with 10% fetal bovine serum (FBS, HyClone) at 37 °C, 5% CO_2_ and 80%-85% humidity (NuAire CO_2_ incubator).

4-hydroxytamoxifen and docetaxel were purchased from Cayman Chemical Company; drug solutions were stored at -70 °C. MCF7/HT cell line was obtained by long-term (for 12 months) cultivation of parental MCF7 cells with antiestrogen 4-hydroxytamoxifen at a concentration of 5 μM. The verification of acquired hormone resistance in MCF7/HT was done by the MTT test.

### The analysis of cell viability

The analysis of cell viability was performed by the MTT test^[28] ^as described earlier in the work^[29]^. The cells were seeded at a density of 4 × 10^4^ cells per well in 24-well plates (Corning) in 900 μL of the medium. The solutions of the tested compounds (4-hydroxytamoxifen, docetaxel) with different concentrations in 100 μL of the medium were added 24 h after the seeding. The cells were cultivated for 72 h, then the medium was removed, and the MTT reagent (3-[4,5-dimethylthiazol-2-yl]-2, 5-diphenyltetrazolium bromide) dissolved in the medium was added to the final concentration of 0.2 mg/mL to each well and incubated for 1 h. After that, the medium was removed, and MTT formazan purple crystals were dissolved in DMSO (300 μL per well). Absorbance was measured at 571 nm with a MultiScan reader (ThermoFisher). The half-maximal inhibitory concentrations (IC_50_) were determined with GraphPad Prism.

### Assessment of ERα activity

MCF7 and MCF7/HT cells were seeded onto a 24-well plate containing a standard cell culture medium with a density of 1.7 × 10^5 ^cells/well. After 24 h, the cells were transfected with the ERE-LUC plasmid containing the luciferase gene under the ERα-dependent promoters^[30]^, and co-transfected with β-galactosidase plasmid. The transfection was performed for 6 h at 37 °C using Lipofectamine 2000 in a medium containing 2% steroid-free serum, then the medium was replaced by a standard medium supplemented with 10% steroid-free serum. The activity of luciferase was assessed according to a Promega protocol using an Infinite M200-Pro, and β-galactosidase activity was assessed using colorimetric assay and MultiScan FC. The internal control values were used to normalize the luciferase/β-galactosidase activities. ERα activity was represented as the mean ± SD for the three independent experiments.

### Immunoblotting and densitometry

Immunoblotting with modifications was performed as described earlier^[31]^. ERα, phospho-Akt, Akt, cleaved PARP, Bcl-2, Bad, phospho-ERKs, ERKs, phospho-S6K, S6K and cyclin D1 expression was evaluated using Cell Signalling Technology (CST) antibodies. Antibodies to α-tubulin (CST) were applied to normalize and control the loading of samples into a gel. Secondary antibodies to rabbit Ig conjugated with horseradish peroxidase were used for the detection (Jackson ImmunoResearch). ECL detection reagents for analysis were prepared according to Mruk and Cheng’s protocol^[32]^. ImageQuant LAS 4000 imager (GE Healthcare) was used to detect signals. Densitometry for the tested proteins/α-tubulin ratio was carried out using ImageJ software.

### Flow cytometry

TUBB3 expression was evaluated by immunofluorescence assay and flow cytometry^[33]^. The cell suspensions at a concentration of 4 × 10^5^ cells/ml were incubated for 1.5 h with primary monoclonal anti-TUBB3 antibody (clone EP1569Y, Abcam, UK) at room temperature in the dark; the suspensions were then incubated for 1.5 h with secondary anti-rabbit antibody conjugated with DyLight®650 (ab98510, Abcam, UK) at + 4°С in the dark. The final dilution for both antibodies was 1:500. Cell fluorescence was measured using the Navios flow cytometer (Beckman Coulter, USA). Two indicators of βIII-tubulin expression were evaluated by FlowJo 10.0.8 (FlowJo, USA): the geometric mean of fluorescence (arb. units) and level of the marker expression (number of specific fluorescent cells). The level of TUBB3 expression was calculated by the Kolmagorov-Smirnov test as the ratio of specifically fluorescent cells to the control cells incubated with secondary antibodies only. The distribution of cells according to fluorescence intensity was visualized using WinMDI 2.9 software.

### Statistical analysis

All data are presented as mean values and standard deviation (mean ± std. deviation). Student’s t-test (GraphPad Prism 9, USA) at *P *< 0.05 was considered to indicate a statistically significant result.

## RESULTS

*In vitro* experiments are an important part of any study in biology and medicine. Their importance is particularly reinforced by modern standards of ethics in science. So, there is a need to analyze the relationship between the doses of a drug given to patients and the concentration of the compound in the culture medium. Are such doses comparable? We first analyzed data from experiments with hormones and their antagonists. The level of 17β-estradiol undergoes significant changes in premenopausal women; usually, the level of this hormone is between 30 and 400 pg/mL^[34-37]^. Thus, we cannot talk about an average level of 17β-estradiol in plasma; in experimental studies, most researchers apply a dose of 17β-estradiol that induces the expression of responsive genes, 10 nM or 2.7 ng/mL^[38,39]^. We see similar trends for antiestrogens, which are used as 17β-estradiol competitors (selective estrogen receptor modulators, SERMs). In the plasma of breast cancer patients who receive tamoxifen, from 391 to 484 ng/mL of the major metabolites of this drug (tamoxifen, N-desmethyltamoxifen, hydroxytamoxifen, endoxifen) were determined^[40,41]^. In *in vitro* experiments, the IC_50_ values of tamoxifen usually exceed the level of 500 ng/mL and were 1-20 µg/mL^[42-44]^. A slightly different situation is observed in the case of chemotherapeutics, in particular docetaxel. The plasma concentration of docetaxel reaches 3737 nM clinically^[45]^, whereas in cell culture experiments, the IC_50_ values do not exceed 10 nM^[46-48]^. Thus, the hormonal drugs are used in higher doses in experiments, while docetaxel is applied in *in vitro* experiments at doses lower than those in the plasma of patients receiving drug treatment. The observations described are consistent with the duration of treatment; hormone therapy is prescribed for long courses (up to 10 years), whereas chemotherapy can be prescribed in short courses due to its high toxicity. In the *in vitro* study presented here, we started from the IC_50_ values for 4-hydroxytamoxifen and assessment of hormonal signaling in obtained resistant cells.

**Figure 1:**
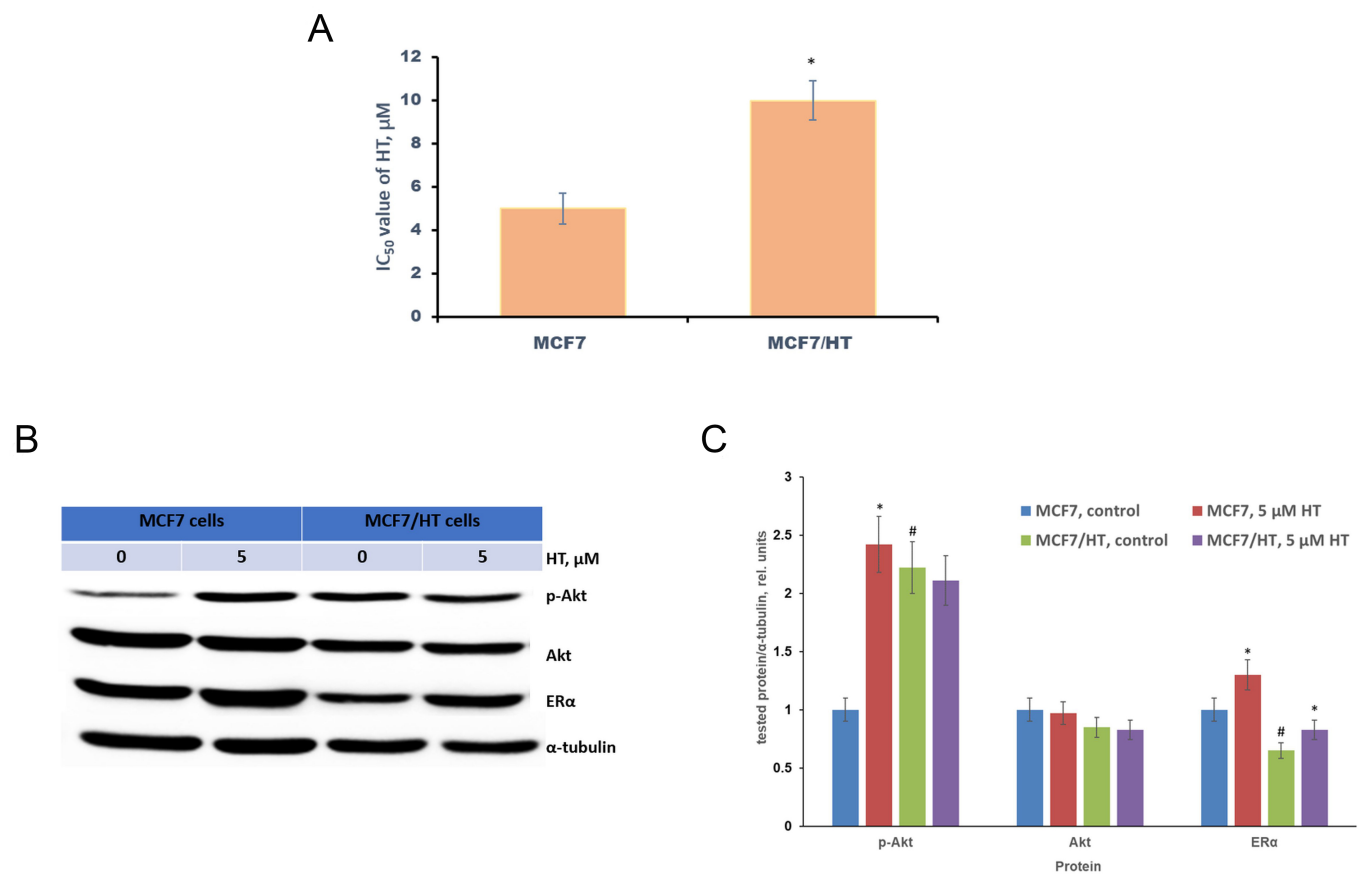
Characteristics of the obtained subline MCF7/HT. (A) - the viability of MCF7/HT and MCF7 cells in the presence of 4-hydroxytamoxifen (HT), *P < 0.05; (B) - Representative immunoblotting images for sensitive MCF7 and resistant MCF/HT cells with antibodies against ERα, p-Akt, and Akt; the cells were treated with 5 µM HT for 24 h and then subjected to immunoblot analysis; (C) - Densitometry for immunoblotting data (*n* = 3) was performed using ImageJ software (Wayne Rasband, NIH). The protocol for analysis was provided by The University of Queensland with the recommendations from the work^[51]^; *P < 0.05 *vs.* corresponding control cells; ^#^*P *< 0.05 *vs.* MCF7 cells.

MCF7/HT was obtained by long-term cultivation of MCF7 breast cancer cells with 4-hydroxytamoxifen. The established MCF7/HT subline has lost sensitivity to 4-hydroxytamoxifen (HT), which was confirmed by the MTT test: the viability of MCF7/HT in the presence of higher concentrations of 4-hydroxytamoxifen compared to MCF7 indicates the developed resistance, the resistance index (IC_50_ of MCF7/HT divided by IC_50_ of MCF7) was 2 [Figure 1A].

It is known that the acquisition of tamoxifen resistance in MCF7 cells is accompanied by the impaired activation of the PI3K/Akt/*PTEN* pathway and down-regulation of ERα^[17,49,50]^. Following the literature data, we have revealed that Akt activity (p-Akt) in MCF7/HT was increased by 2.2-fold [Figure 1B and C]. Moreover, ERα expression was decreased by 1.5-fold.

**Figure 2:**
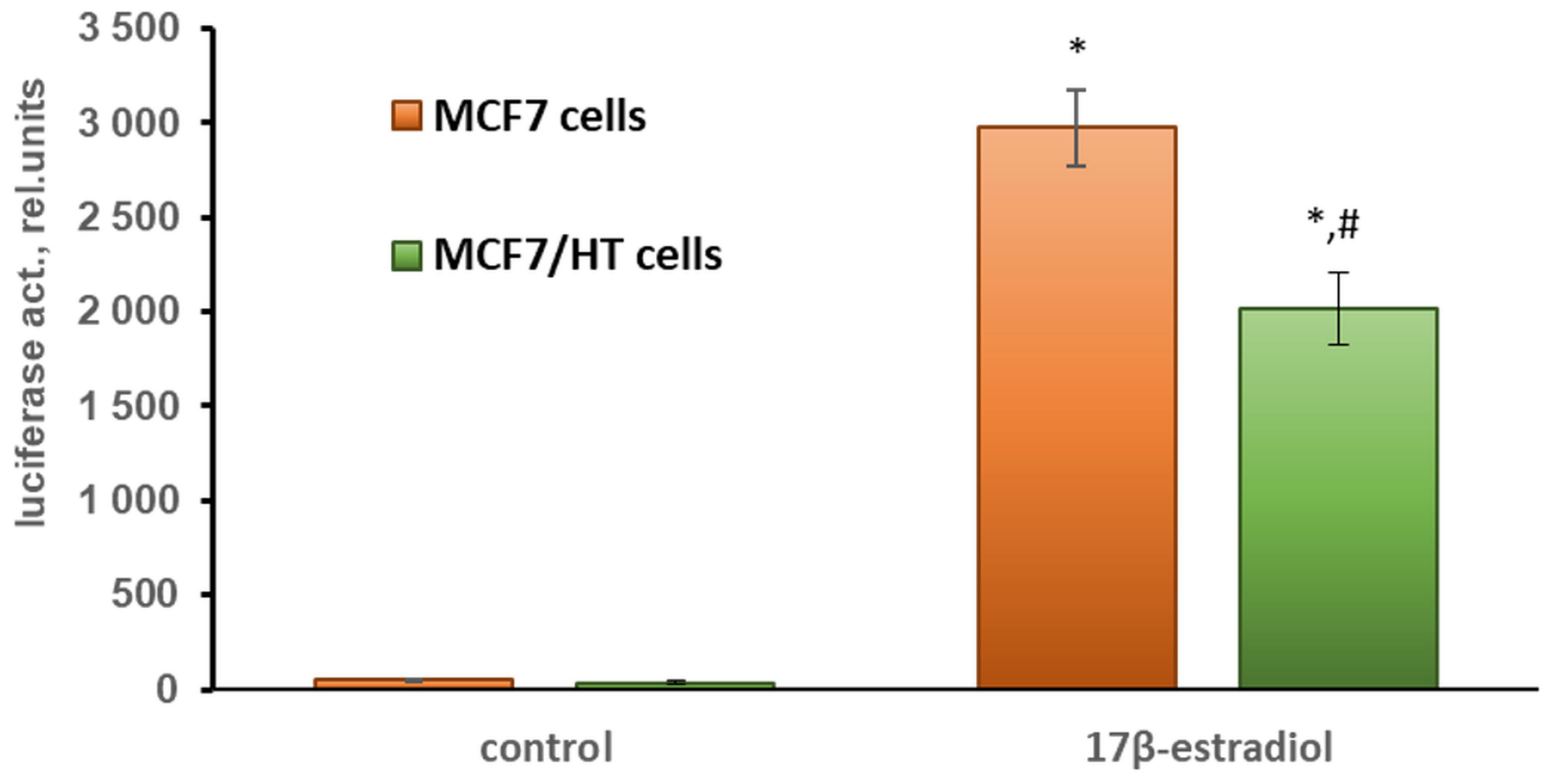
17β-estradiol-induced activity of ERα in MCF7 cells assessed by gene reporter assay; *P < 0.05 *vs.* control cells; ^#^*P *< 0.05 *vs.* MCF7 cells treated with 10 nM 17β-estradiol. The mean values of three independent experiments are shown.

To assess ERα activity, the cells were transfected with the plasmids containing the luciferase gene under the estrogen receptor α-dependent promoters (ERE-LUC). The activity of ERα was induced by its physiological ligand, 17β-estradiol^[30,52]^. As can be seen in Figure 2, the induced activity of the estrogen receptor α in MCF7/HT cells was decreased by 1.5-fold (*P *< 0.05) when compared to that in parental cells. This indicates a partial loss of hormonal dependence of the breast cancer cells after long-term incubation with 4-hydroxytamoxifen.

**Figure 3:**
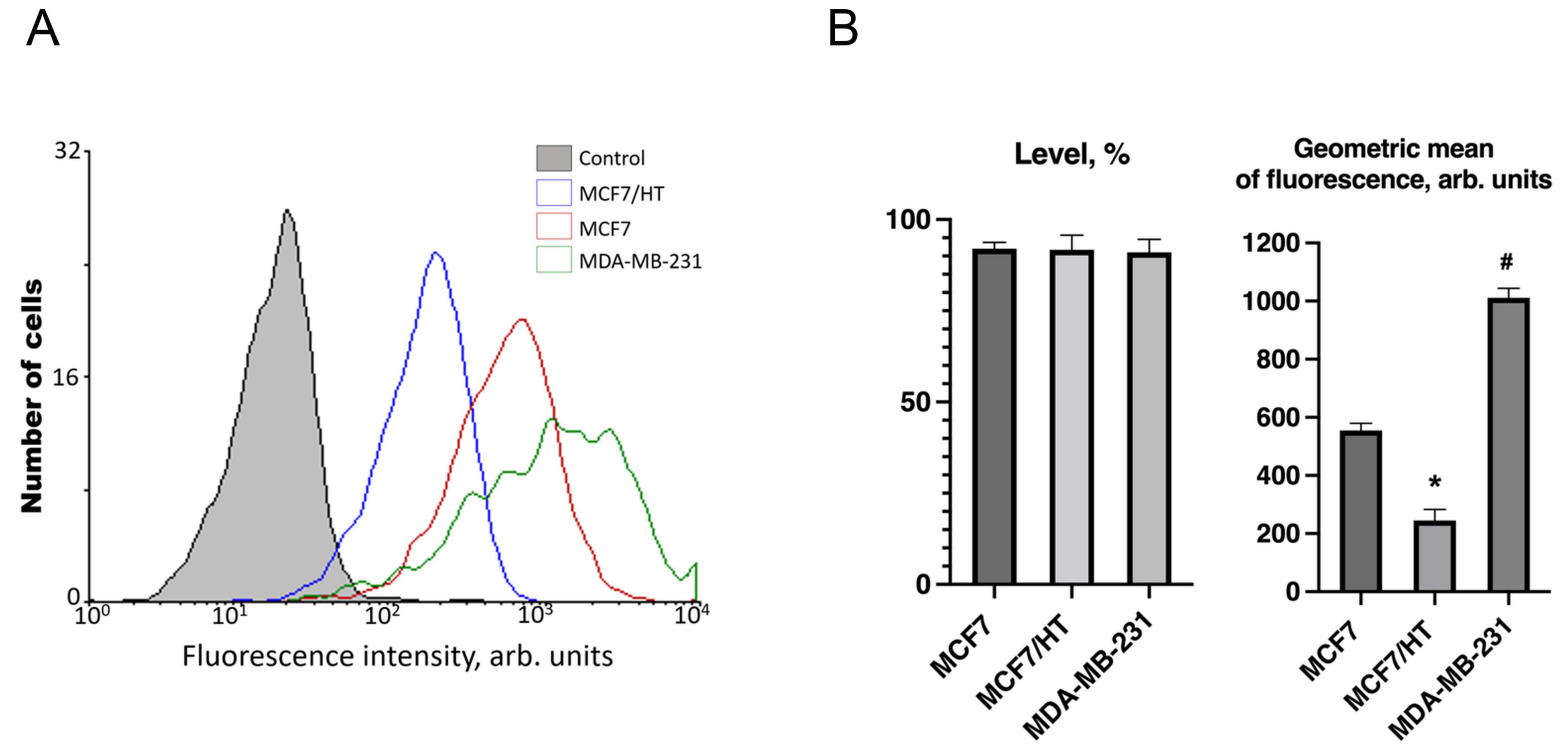
βIII-tubulin expression in breast cancer cell lines. (A) - Representative diagram of βIII-tubulin expression. The abscissa is the intensity of specific fluorescence in the channel of an FL6 flow cytometer (arb. units); the ordinate axis is the number of cells. Control - autofluorescence of samples; (B) - A difference in TUBB3 expression was revealed between the cell lines, and the intensity of the marker expression increased in the following order (*n *= 3): MCF7/HT < MCF7 < MDA-MB-231 (approximately, 1:2:4). *P < 0.05 *vs.* MCF7 and MDA-MB-231, ^#^*P *< 0.05 *vs.* MCF7 and MCF7/HT.

In subsequent experiments, we used MDA-MB-231 cells which are triple-negative; these cells are hormone-resistant *de novo*^[53]^. Analysis of βIII-tubulin (TUBB3) expression revealed the following trends: the level of TUBB3 expression was high and the same in all three studied cell lines, while the geometric mean of the marker expression differed dramatically [Figure 3].

Higher expression of TUBB3 was detected in triple-negative breast cancer MDA-MB-231 cells compared to hormone-responsive MCF7 cells [Figure 3]. The lowest TUBB3 expression was found in hormone-resistant MCF7/HT cells; the geometric mean of the marker was reduced by more than 2 and 4 times in comparison with MCF7 and MDA-MB-231 cells, respectively. Figure 3 shows one of the typical experiments for the evaluation of TUBB3 protein expression in the studied cell lines.

**Figure 4:**
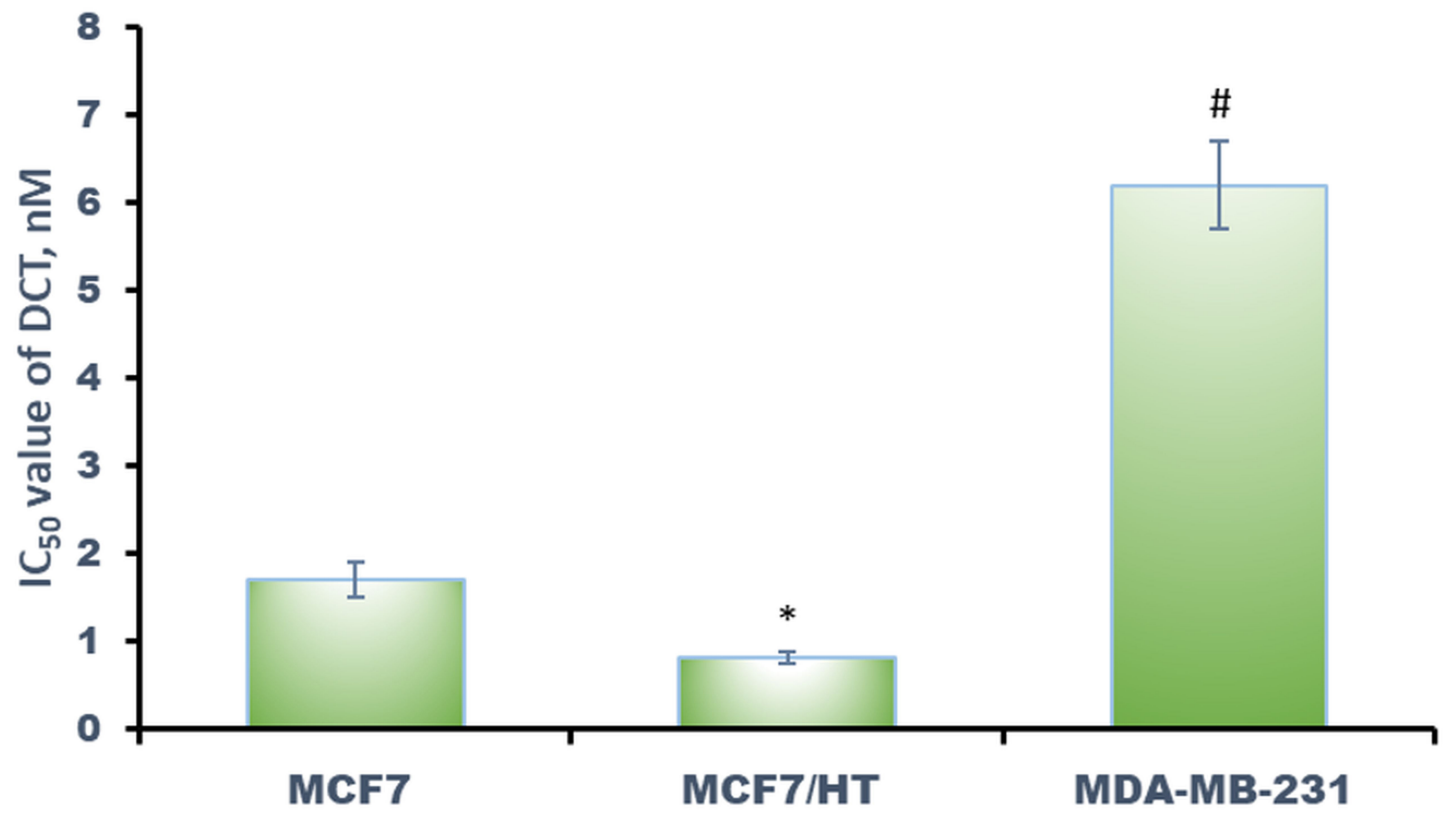
Effects of docetaxel (DCT) against breast cancer cells. *P < 0.05 *vs.* MCF7 and MDA-MB-231, ^#^*P *< 0.05 *vs.* MCF7 and MCF7/HT. The results for three independent experiments are shown.

High TUBB3 expression strongly correlated (*P *< 0.05) with docetaxel resistance: IC_50_ value of docetaxel for MDA-MB-231 cells was greater than that for MCF7 cells, hormone-resistant MCF7/HT cells were the most sensitive to the drug [Figure 4].

**Figure 5:**
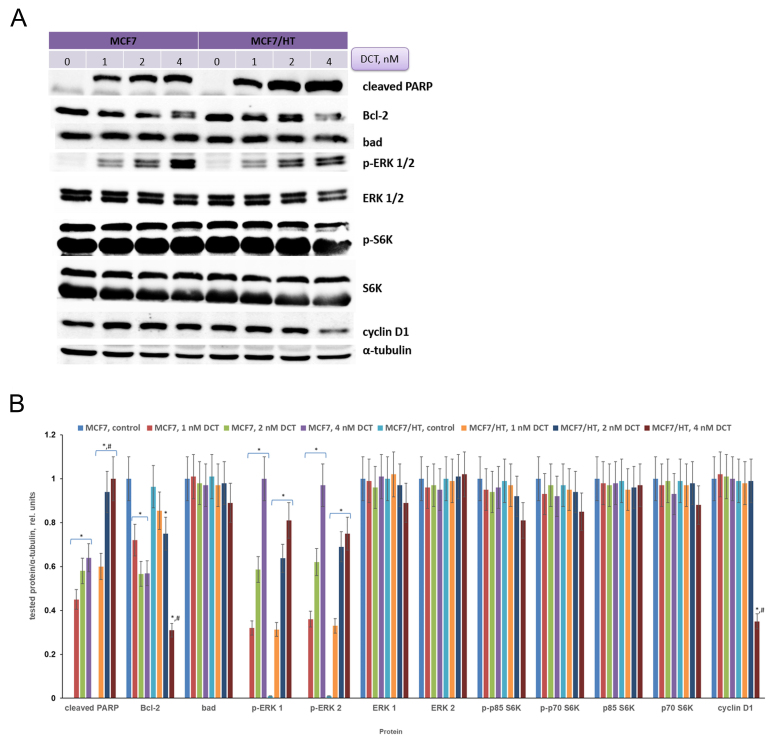
Effects of docetaxel on signaling in MCF7 and MCF7/HT cells. (A) - Representative immunoblotting images of MCF7 and MCF/HT cells with antibodies against cleaved PARP, Bcl-2, Bad, p-ERK 1/2, ERK 1/2, p-S6K, S6K, and cyclin D1; the cells were treated with docetaxel (DCT) for 24 h and then subjected to immunoblot analysis. Antibodies against α-tubulin were used as loading control. (B) - Densitometry for immunoblotting data (*n* = 3); *P < 0.05 *vs.* corresponding control cells; ^#^*P *< 0.05 *vs.* corresponding DCT-treated MCF7 cells.

In subsequent experiments, changes in signaling pathways induced by docetaxel were analyzed. MCF7 and MCF7/HT cells were treated with docetaxel and then protein expression was analyzed in the obtained samples. There are many ways of detecting apoptosis in cells, one of such approaches is to determine the level of cleaved PARP (poly (ADP-ribose) polymerase)^[54]^. The cleaved PARP may be considered a marker of apoptosis. As shown in Figure 5, incubation of cells with the drug leads to a dose-dependent increase in cleaved PARP expression. This indicates that docetaxel causes apoptosis. It is important to note that the accumulation of cleaved PARP is more pronounced in DCT-treated resistant cells (1.6-fold). Regulation of cell death pathways occurs with the participation of proteins from various families. The balance between proapoptotic and antiapoptotic proteins is usually observed in unchanged cells. Activation of antiapoptotic pathways is often detected in tumor cells. The expression of the antiapoptotic factor Bcl-2 was analyzed in MCF7 and MCF7/HT cells. Docetaxel caused a decrease in Bcl-2 expression in both cell lines, but the observed effects were more prominent in the resistant ones treated with 4 nM DCT (1.8-fold). Analysis of the expression of the proapoptotic protein Bad showed no differences between sensitive and resistant cells.

Docetaxel caused significant activation of ERKs in both cell lines (*P *< 0.05) and its effect was dose-dependent, as shown in Figure 5. The S6 kinase (S6K) is one of the effectors of the mammalian Target Of Rapamycin (mTOR); S6K regulates protein synthesis and cell growth. The overexpressed S6K was found in a variety of tumors and correlated to poor prognosis in cancers^[55]^. Amaral *et al.* described that S6Ks isoforms contribute to migration, viability, resistance to docetaxel and tumor formation of cancer cells^[55]^. We tested whether S6K is involved in the cell response to docetaxel. As shown in Figure 5, there were no differences in S6K expression between MCF7 and MCF7/HT cells. High activity of S6K was detected in all obtained samples, while docetaxel did not alter its activity or expression in both cell lines [Figure 5]. Intriguingly, the expression of the cell cycle regulator cyclin D1 decreased (2.8-fold) only in resistant cells after docetaxel treatment, while this marker was unchanged in parental MCF7 cells.

## DISCUSSION

The interest in βIII-tubulin in cancer research is related to its role in drug tolerance of various tumours, particularly to taxanes, that block tubulin depolymerization, thereby increasing the content of polymerized tubulin and arresting cellular functions. Overexpression of βIII-tubulin was found in many tumours. TUBB3 expression was associated with a poor response to various drugs, including docetaxel, paclitaxel, vinca alkaloids, cisplatin, etoposide, and doxorubicin. βIII-tubulin overexpression was associated with a poor response to microtubule-targeting agents and a shorter overall and progression-free survival in various cancers, including bladder, lung, ovarian, breast, prostate, and rectal cancers. Moreover, βIII-tubulin expression was positively correlated with lymphatic metastasis and tumor differentiation^[23]^. TUBB3 is also involved in docetaxel (DCT) and cabazitaxel (CBZ) resistance. LY294002, a PI3K inhibitor, re-sensitized DCT-resistant cell lines to docetaxel and CBZ-resistant cell lines to cabazitaxel (CBZ). The combination of DCT/CBZ and LY294002 could be potential strategy for the treatment of prostate cancer^[56]^.

Several approaches have been developed to enhance the action of docetaxel. In preclinical projects, researchers use specific nucleotides against signaling RNAs to modulate the action of docetaxel. Dr Razi Soofiyani *et al. *described very interesting experiments with siRNA-mediated silencing of CIP2A^[57,58]^. CIP2A silencing enhanced the sensitivity of cancer cells to DCT by strengthening drug-induced cell growth inhibition and apoptosis. The authors concluded that CIP2A silencing may potentiate the antiproliferative effects of docetaxel and this might be a promising therapeutic approach in prostate cancer treatment. cMET is considered as a target in the work by Dr Majidi Zolbanin *et al.*^[59]^. The mucin1 aptamer-conjugated chitosan nanoparticles, containing docetaxel and cMET siRNA, were suggested by authors for the treatment of mucin1-positive metastatic breast cancers.

The molecular pathway of ERKs plays a crucial role in the growth and death of normal and cancer cells^[60]^. Depending on the stimulus, activity of ERKs mediates various antiproliferative signals, such as apoptosis and autophagy^[61-63]^. Chemotherapeutic and other DNA damage agents, including etoposide, doxorubicin, ionizing radiation and ultraviolet irradiation (UV), activate protein kinase ERK1/2 in various cells^[61,64]^. Previously published data are in good agreement with our observations on ERK activity in cells after DCT treatment. Lucie Chauvin *et al.* discuss the possible role of ERK in maintaining the survival of docetaxel-treated cells in the work^[65]^. Authors demonstrated that docetaxel supports a survival signaling pathway through a mechanism depending on PKC and ERKs in the MDA-MB-231 breast cancer cells. Thus, the use of combinations of docetaxel with ERK inhibitors could be a promising strategy for future studies of breast cancers.

The relationship between βIII-tubulin expression and taxane-resistance of tumours is being extensively studied. For example, Maahs *et al.* have studied βIII-tubulin expression as a predictor of resistance in patients with metastatic castration-resistant prostate cancer and have revealed that patients with a high expression of TUBB3 had a lower survivability and worse response rates to docetaxel as indicated by a 10% or greater decrease in prostate-specific antigen (PSA) compared to a 50% or more decrease in patients with a low βIII-tubulin who have a better response rate to docetaxel^[66]^.

De Donato *et al.* have considered TUBB3 as a gateway for survival PIM1 signals^[67]^. The cells are exposed to microenvironmental stressors and PIM1 was incorporated into the cytoskeleton through GBP1 and βIII-tubulin, which ultimately leads to drug resistance. Moreover, De Donato *et al.* have found a statistically significant up-regulation of class III beta-tubulin in the paclitaxel-resistant ovarian tumors^[67]^. Similarly, Roque *et al.* have found that TUBB3 overexpression in clear cell carcinoma of the ovary discriminates poor prognosis. High TUBB3 expression is a marker for sensitivity to patupilone and may contribute to resistance to paclitaxel^[68]^.

The results of our study are consistent with the evidence described above: the cells with the highest βIII-tubulin expression (MDA-MB-231) were resistant to docetaxel and the cells with the lowest βIII-tubulin expression (MCF7/HT) were sensitive to docetaxel. Several researches show that βIII-tubulin expression in cancer cells is regulated by hormones. Saussede-Aim *et al.* showed that 17β-estradiol exposure causes an up-regulation of βIII-tubulin in ERα-positive MCF7 breast cancer cells, and estrogen receptor modulators (e.g. tamoxifen) reduce the βIII-tubulin level in ERα-positive breast cancer cells, but did not affect the βIII-tubulin level in ERα-negative MDA-MB-231 cells^[69]^. This mentioned observation is in good agreement with our data. We have shown that estrogen receptor α activity is decreased in the hormone-resistant cells, and the hormone dependence of the cells decreases accordingly. A decrease in βIII-tubulin expression may be associated with these changes in hormone signaling of cancer cells. There is also evidence that androgens modulate TUBB3 expression: in prostate cancer cells and patient tumors, androgen ablation correlates with high TUBB3 levels^[70]^. In another work, an increase in βIII-tubulin was revealed in androgen-starved and androgen receptors knockdown human prostate adenocarcinoma cells LNCaP^[71]^.

Consistent with these facts, MCF7/HT in our study had the lowest activity of ERα along with the lowest βIII-tubulin level; on the contrary, MDA-MB-231 with no ERα at all had the highest βIII-tubulin level. Docetaxel affected the expression of a number of signaling proteins in MCF7 and MCF7/HT breast cancer cells. The accumulation of cleaved PARP (a marker of apoptosis) and Bcl-2 downregulation were more pronounced in resistant cells. Moreover, the expression of cyclin D1 decreased only in resistant cells after docetaxel treatment, while this marker was unchanged in parental MCF7 cells. Interestingly, according to several clinical trials, a high level of TUBB3 is associated with negativity for estrogen and progesterone receptors in breast cancer patients^[72,73]^ and, as a result, with worse disease-free and overall survival^[73,74]^.

In conclusion, high Akt activity (a 2.2-fold increase) and decreased activity of the estrogen receptor α (1.5-fold) were found in established hormone-resistant MCF7/HT cells. It is intriguing that the expression of TUBB3, metastasis-associated tubulin, was lowered in the hormone-resistant cells. The hormone-resistant cells were characterized by high sensitivity to tubulin polymerization inhibitor docetaxel, belonging to taxanes. The significant accumulation of cleaved PARP (1.6-fold) and Bcl-2 downregulation (1.8-fold) were revealed in DCT-treated MCF7/HT cells. Thus, further development of taxane-based chemotherapy for hormone-resistant cancer with low TUBB3 expression looks highly promising.

## DECLARATIONS

### Availability of data and materials

The material used and/or analyzed during the present study are available from the corresponding author upon reasonable request.

### Copyright

© The Author(s) 2023.

## References

[1] Trayes KP, Cokenakes SEH (2021). Breast cancer treatment. Am Fam Physician.

[2] Clarke R, Jones BC, Sevigny CM, Hilakivi-Clarke LA, Sengupta S (2021). Experimental models of endocrine responsive breast cancer: strengths, limitations, and use. Cancer Drug Resist.

[3] Bai Z, Gust R (2009). Breast cancer, estrogen receptor and ligands. Arch Pharm (Weinheim).

[4] (2016). Marchi T, Foekens JA, Umar A, Martens JW. Endocrine therapy resistance in estrogen receptor (ER)-positive breast cancer. Drug Discov Today.

[5] Fisher B, Redmond C, Brown A (1983). Influence of tumor estrogen and progesterone receptor levels on the response to tamoxifen and chemotherapy in primary breast cancer. J Clin Oncol.

[6] Jordan VC (2021). 50th anniversary of the first clinical trial with ICI 46,474 (tamoxifen): then what happened?. Endocr Relat Cancer.

[7] Robertson JF (2001). ICI 182,780 (Fulvestrant)--the first oestrogen receptor down-regulator--current clinical data. Br J Cancer.

[8] Gaudet HM, Cheng SB, Christensen EM, Filardo EJ (2015). The G-protein coupled estrogen receptor, GPER: the inside and inside-out story. Mol Cell Endocrinol.

[9] Zhang J, Zhou C, Jiang H (2017). ZEB1 induces ER-α promoter hypermethylation and confers antiestrogen resistance in breast cancer. Cell Death Dis.

[10] Toy W, Shen Y, Won H (2013). ESR1 ligand-binding domain mutations in hormone-resistant breast cancer. Nat Genet.

[11] Wang ZY, Yin L (2015). Estrogen receptor alpha-36 (ER-α36): a new player in human breast cancer. Mol Cell Endocrinol.

[12] Cottu PH (2017). [Systemic neoadjuvant therapy of luminal breast cancer in 2016]. Bull Cancer.

[13] André F, Bachelot T, Campone M (2013). Targeting FGFR with dovitinib (TKI258): preclinical and clinical data in breast cancer. Clin Cancer Res.

[14] Fan P, Jordan VC (2019). New insights into acquired endocrine resistance of breast cancer. Cancer Drug Resist.

[15] Hyder T, Marti JLG, Nasrazadani A, Brufsky AM (2021). Statins and endocrine resistance in breast cancer. Cancer Drug Resist.

[16] Miller TW, Hennessy BT, González-Angulo AM (2010). Hyperactivation of phosphatidylinositol-3 kinase promotes escape from hormone dependence in estrogen receptor-positive human breast cancer. J Clin Invest.

[17] Bostner J, Karlsson E, Pandiyan MJ (2013). Activation of Akt, mTOR, and the estrogen receptor as a signature to predict tamoxifen treatment benefit. Breast Cancer Res Treat.

[18] Aleskandarany MA, Rakha EA, Ahmed MA, Powe DG, Ellis IO, Green AR (2011). Clinicopathologic and molecular significance of phospho-Akt expression in early invasive breast cancer. Breast Cancer Res Treat.

[19] Spears M, Cunningham CA, Taylor KJ (2012). Proximity ligation assays for isoform-specific Akt activation in breast cancer identify activated Akt1 as a driver of progression. J Pathol.

[20] Gudimchuk NB, McIntosh JR (2021). Regulation of microtubule dynamics, mechanics and function through the growing tip. Nat Rev Mol Cell Biol.

[21] Di Bartolomeo M, Raimondi A, Cecchi F (2021). Association of high TUBB3 with resistance to adjuvant docetaxel-based chemotherapy in gastric cancer: translational study of ITACA-S. Tumori.

[22] Duly AMP, Kao FCL, Teo WS, Kavallaris M (2022). βIII-tubulin gene regulation in health and disease. Front Cell Dev Biol.

[23] Kanakkanthara A, Miller JH (2021). βIII-tubulin overexpression in cancer: causes, consequences, and potential therapies. Biochim Biophys Acta Rev Cancer.

[24] Imran M, Saleem S, Chaudhuri A, Ali J, Baboota S (2020). Docetaxel: an update on its molecular mechanisms, therapeutic trajectory and nanotechnology in the treatment of breast, lung and prostate cancer. J Drug Deliv Sci Technol.

[25] Raza F, Zafar H, You X, Khan A, Wu J, Ge L (2019). Cancer nanomedicine: focus on recent developments and self-assembled peptide nanocarriers. J Mater Chem B.

[26] Yu Y, Qiu L (2016). Optimizing particle size of docetaxel-loaded micelles for enhanced treatment of oral epidermoid carcinoma. Nanomedicine.

[27] Gao X, Zhang J, Huang Z (2017). Reducing interstitial fluid pressure and inhibiting pulmonary metastasis of breast cancer by gelatin modified cationic lipid nanoparticles. ACS Appl Mater Interf.

[28] Iselt M, Holtei W, Hilgard P (1989). The tetrazolium dye assay for rapid in vitro assessment of cytotoxicity. Arzneimittelforschung.

[29] Volkova YA, Antonov YS, Komkov AV (2016). Access to steroidal pyridazines via modified thiohydrazides. RSC Adv.

[30] Reid G, Hübner MR, Métivier R (2003). Cyclic, Proteasome-mediated turnover of unliganded and liganded ERα on responsive promoters is an integral feature of estrogen signaling. Molecular Cell.

[31] Scherbakov AM, Lobanova YS, Shatskaya VA, Onopchenko OV, Gershtein ES, Krasil'nikov MA (2006). Activation of mitogenic pathways and sensitization to estrogen-induced apoptosis: two independent characteristics of tamoxifen-resistant breast cancer cells?. Breast Cancer Res Treat.

[32] Mruk DD, Cheng CY (2011). Enhanced chemiluminescence (ECL) for routine immunoblotting: an inexpensive alternative to commercially available kits. Spermatogenesis.

[33] Bogush TA, Maiak MA, Saprykina NS (2021). Experimental verification on the hypothesis about the possibility of molecular diagnostics of local tumor spread on the lewis lung carcinoma model. Moscow Univ Chem Bull.

[34] Bergemann N, Mundt C, Parzer P (2005). Plasma concentrations of estradiol in women suffering from schizophrenia treated with conventional versus atypical antipsychotics. Schizophr Res.

[35] Hassan LS, Monson RS, Danielson KK (2017). Oestradiol levels may differ between premenopausal women, ages 18-50, with type 1 diabetes and matched controls. Diabetes Metab Res Rev.

[36] Angsuwathana S, Tanmahasamut P, Rattanachaiyanont M (2006). Serum follicle stimulating hormone and estradiol in peri/postmenopausal women attending Siriraj Menopause Clinic: a retrospective study. J Med Assoc Thai.

[37] Huitrón-Bravo G, Denova-Gutiérrez E, Talavera JO (2016). Levels of serum estradiol and lifestyle factors related with bone mineral density in premenopausal Mexican women: a cross-sectional analysis. BMC Musculoskelet Disord.

[38] Nardulli AM, Romine LE, Carpo C, Greene GL, Rainish B (1996). Estrogen receptor affinity and location of consensus and imperfect estrogen response elements influence transcription activation of simplified promoters. Mol Endocrinol.

[39] Rato AG, Pedrero JG, Martinez MA, del Rio B, Lazo PS, Ramos S (1999). Melatonin blocks the activation of estrogen receptor for DNA binding. FASEB J.

[40] Bobin-Dubigeon C, Campone M, Rossignol E, Salaun E, Amiand MB, Bard JM (2019). New UPLC-MS/MS assay for the determination of tamoxifen and its metabolites in human plasma, application to patients. Future Sci OA.

[41] Woo HI, Lee SK, Kim J (2017). Variations in plasma concentrations of tamoxifen metabolites and the effects of genetic polymorphisms on tamoxifen metabolism in Korean patients with breast cancer. Oncotarget.

[42] Hassan F, Mohammed G, El-hiti GA, Alshanon A, Yousif E (2018). Cytotoxic effects of tamoxifen in breast cancer cells. J Unexplored Med Data.

[43] Kuznetsov YV, Levina IS, Scherbakov AM (2018). New estrogen receptor antagonists. 3,20-Dihydroxy-19-norpregna-1, 3, 5(10)-trienes: synthesis, molecular modeling, and biological evaluation. Eur J Med Chem.

[44] Seeger H, Huober J, Wallwiener D, Mueck AO (2004). Inhibition of human breast cancer cell proliferation with estradiol metabolites is as effective as with tamoxifen. Horm Metab Res.

[45] Brunsvig PF, Andersen A, Aamdal S, Kristensen V, Olsen H (2007). Pharmacokinetic analysis of two different docetaxel dose levels in patients with non-small cell lung cancer treated with docetaxel as monotherapy or with concurrent radiotherapy. BMC Cancer.

[46] Tsakalozou E, Eckman AM, Bae Y (2012). Combination effects of docetaxel and Doxorubicin in hormone-refractory prostate cancer cells. Biochem Res Int.

[47] Gupta S, Weston A, Bearrs J (2016). Reversible lysine-specific demethylase 1 antagonist HCI-2509 inhibits growth and decreases c-MYC in castration- and docetaxel-resistant prostate cancer cells. Prostate Cancer Prostatic Dis.

[48] Samadi N, Ghanbari P, Mohseni M (2014). Combination therapy increases the efficacy of docetaxel, vinblastine and tamoxifen in cancer cells. J Cancer Res Ther.

[49] García-Becerra R, Santos N, Díaz L, Camacho J (2012). Mechanisms of resistance to endocrine therapy in breast cancer: focus on signaling pathways, miRNAs and genetically based resistance. Int J Mol Sci.

[50] Ali S, Rasool M, Chaoudhry H (2016). Molecular mechanisms and mode of tamoxifen resistance in breast cancer. Bioinformation.

[51] Taylor SC, Berkelman T, Yadav G, Hammond M (2013). A defined methodology for reliable quantification of Western blot data. Mol Biotechnol.

[52] Basappa B, Chumadathil Pookunoth B, Shinduvalli Kempasiddegowda M, Knchugarakoppal Subbegowda R, Lobie PE, Pandey V (2021). Novel biphenyl amines inhibit oestrogen receptor (ER)-α in ER-positive mammary carcinoma cells. Molecules.

[53] Chavez KJ, Garimella SV, Lipkowitz S (2010). Triple negative breast cancer cell lines: one tool in the search for better treatment of triple negative breast cancer. Breast Dis.

[54] Chaitanya GV, Steven AJ, Babu PP (2010). PARP-1 cleavage fragments: signatures of cell-death proteases in neurodegeneration. Cell Commun Signal.

[55] Amaral CL, Freitas LB, Tamura RE (2016). S6Ks isoforms contribute to viability, migration, docetaxel resistance and tumor formation of prostate cancer cells. BMC Cancer.

[56] Sekino Y, Han X, Kawaguchi T (2019). TUBB3 reverses resistance to docetaxel and cabazitaxel in prostate cancer. Int J Mol Sci.

[57] (2017). Razi Soofiyani S, Mohammad Hoseini A, Mohammadi A, Khaze Shahgoli V, Baradaran B, Hejazi MS. siRNA-mediated silencing of CIP2A enhances docetaxel activity against PC-3 prostate cancer cells. Adv Pharm Bull.

[58] Razi Soofiyani S, Baradaran B, Lotfipour F, Kazemi T, Mohammadnejad L (2013). Gene therapy, early promises, subsequent problems, and recent breakthroughs. Adv Pharm Bull.

[59] Majidi Zolbanin N, Jafari R, Majidi J (2018). Targeted Co-delivery of docetaxel and cMET siRNA for treatment of mucin1 overexpressing breast cancer cells. Adv Pharm Bull.

[60] Sale MJ, Balmanno K, Cook SJ (2019). Resistance to ERK1/2 pathway inhibitors; sweet spots, fitness deficits and drug addiction. Cancer Drug Resist.

[61] Cagnol S, Chambard JC (2010). ERK and cell death: mechanisms of ERK-induced cell death - apoptosis, autophagy and senescence. FEBS J.

[62] Subramaniam S, Unsicker K (2010). ERK and cell death: ERK1/2 in neuronal death. FEBS J.

[63] Llorens F, Miró FA, Casañas A (2004). Unbalanced activation of ERK1/2 and MEK1/2 in apigenin-induced HeLa cell death. Exp Cell Res.

[64] Tang D, Wu D, Hirao A (2002). ERK activation mediates cell cycle arrest and apoptosis after DNA damage independently of p53. J Biol Chem.

[65] Chauvin L, Goupille C, Blanc C (2016). Long chain n-3 polyunsaturated fatty acids increase the efficacy of docetaxel in mammary cancer cells by downregulating Akt and PKCε/δ-induced ERK pathways. Biochim Biophys Acta.

[66] Maahs L, Sanchez BE, Gupta N (2019). Class III β-tubulin expression as a predictor of docetaxel-resistance in metastatic castration-resistant prostate cancer. PLoS One.

[67] De Donato M, Mariani M, Petrella L (2012). Class III β-tubulin and the cytoskeletal gateway for drug resistance in ovarian cancer. J Cell Physiol.

[68] Roque DM, Bellone S, Buza N (2013). Class III β-tubulin overexpression in ovarian clear cell and serous carcinoma as a marker for poor overall survival after platinum/taxane chemotherapy and sensitivity to patupilone. Am J Obstet Gynecol.

[69] Saussede-Aim J, Matera EL, Ferlini C, Dumontet C (2009). Beta3-tubulin is induced by estradiol in human breast carcinoma cells through an estrogen-receptor dependent pathway. Cell Motil Cytoskeleton.

[70] Terry S, Ploussard G, Allory Y (2009). Increased expression of class III beta-tubulin in castration-resistant human prostate cancer. Br J Cancer.

[71] Wright ME, Tsai MJ, Aebersold R (2003). Androgen receptor represses the neuroendocrine transdifferentiation process in prostate cancer cells. Mol Endocrinol.

[72] Lebok P, Öztürk M, Heilenkötter U (2016). High levels of class III β-tubulin expression are associated with aggressive tumor features in breast cancer. Oncol Lett.

[73] Pentheroudakis G, Batistatou A, Kalogeras KT (2011). Prognostic utility of β-tubulin isotype III and correlations with other molecular and clinicopathological variables in patients with early breast cancer: a translational Hellenic Cooperative Oncology Group (HeCOG) study. Breast Cancer Res Treat.

[74] Hugh J, Hanson J, Cheang MC (2009). Breast cancer subtypes and response to docetaxel in node-positive breast cancer: use of an immunohistochemical definition in the BCIRG 001 trial. J Clin Oncol.

